# Comparative analysis of chronic neuropathic pain and pain assessment in companion animals and humans

**DOI:** 10.3389/fvets.2024.1520043

**Published:** 2024-12-10

**Authors:** Rell L. Parker

**Affiliations:** Department of Small Animal Clinical Sciences, VA-MD College of Veterinary Medicine, Virginia Tech, Blacksburg, VA, United States

**Keywords:** chronic pain, neuropathic pain, neurology, translational medicine, canine

## Abstract

Chronic neuropathic pain is underdiagnosed in companion animals. This paper will review the definition of pain and how classification and grading of neuropathic pain can be applied from human to veterinary medicine to increase the recognition of and the confidence in a neuropathic pain diagnosis. The mechanisms of nociception and the pathophysiology of the sensory systems that underlie the transition to chronic pain are described. Potential future methods for diagnosis and treatment of neuropathic pain in veterinary medicine are considered, utilizing the theoretical framework of pain behavior from humans and rodents. By discussing the current state of pain diagnosis in companion animals and increasing the recognition of chronic neuropathic pain, the goal is to increase understanding of chronic neuropathic pain in daily clinical practice and to aid the development of methods to diagnose and treat neuropathic pain.

## 1 Introduction

Pain diagnosis and management in human medicine is challenging, but in veterinary medicine, additional factors compound this inherent challenge. Definitions of pain used in human medicine can be applied to veterinary medicine (1). The definition of pain from the International Association for the Study of Pain (IASP) is: “the unpleasant sensory and emotional experience associated with, or resembling that associated with, actual or potential tissue damage” (2). The IASP recognizes that the lack of verbal description does not remove the possibility that pain is experienced and therefore recognizes that pain occurs in non-human animals (2). In companion animals, veterinarians and veterinary team members detect and measure pain by observation, examination, and obtaining a through history from pet-owners.

Differentiating pain from anxiety, cognitive dysfunction, or other behavioral disorders is an important aspect of diagnosing pain in companion animals (3–5). Once pain is diagnosed, the goal is to determine the source and type of the pain, for example, separating neuropathic and musculoskeletal pain (6). In veterinary species, as in human medicine, there are limited effective treatment options for neuropathic pain (7, 8). New technologies for diagnosis and treatment of pain in veterinary and human medicine are under development. However, large gaps remain in our understanding of pain pathophysiology in all species.

The diagnosis and measurement of chronic pain in clinical companion animal practice can be improved by refining pain classification, quantifying signs of pain, attempting to separate signs of pain and anxiety, and developing markers of pain that are not based on clinical examination findings (1). Although several types of pain affect both companion animals and humans, we will focus here on neuropathic pain in companion animals, which is likely underdiagnosed and may be better understood by considering neuropathic pain classification and diagnosis in humans (1, 6, 9). This review will discuss the physiology and pathophysiology of chronic neuropathic pain, challenges and opportunities in veterinary practice, and methods to combine current clinical practices in veterinary medicine with advances in human and rodent pain management to better detect and treat pain in our veterinary species.

## 2 Classification of neuropathic pain in humans and probable sources of neuropathic pain in companion animals

**Figure 1:**
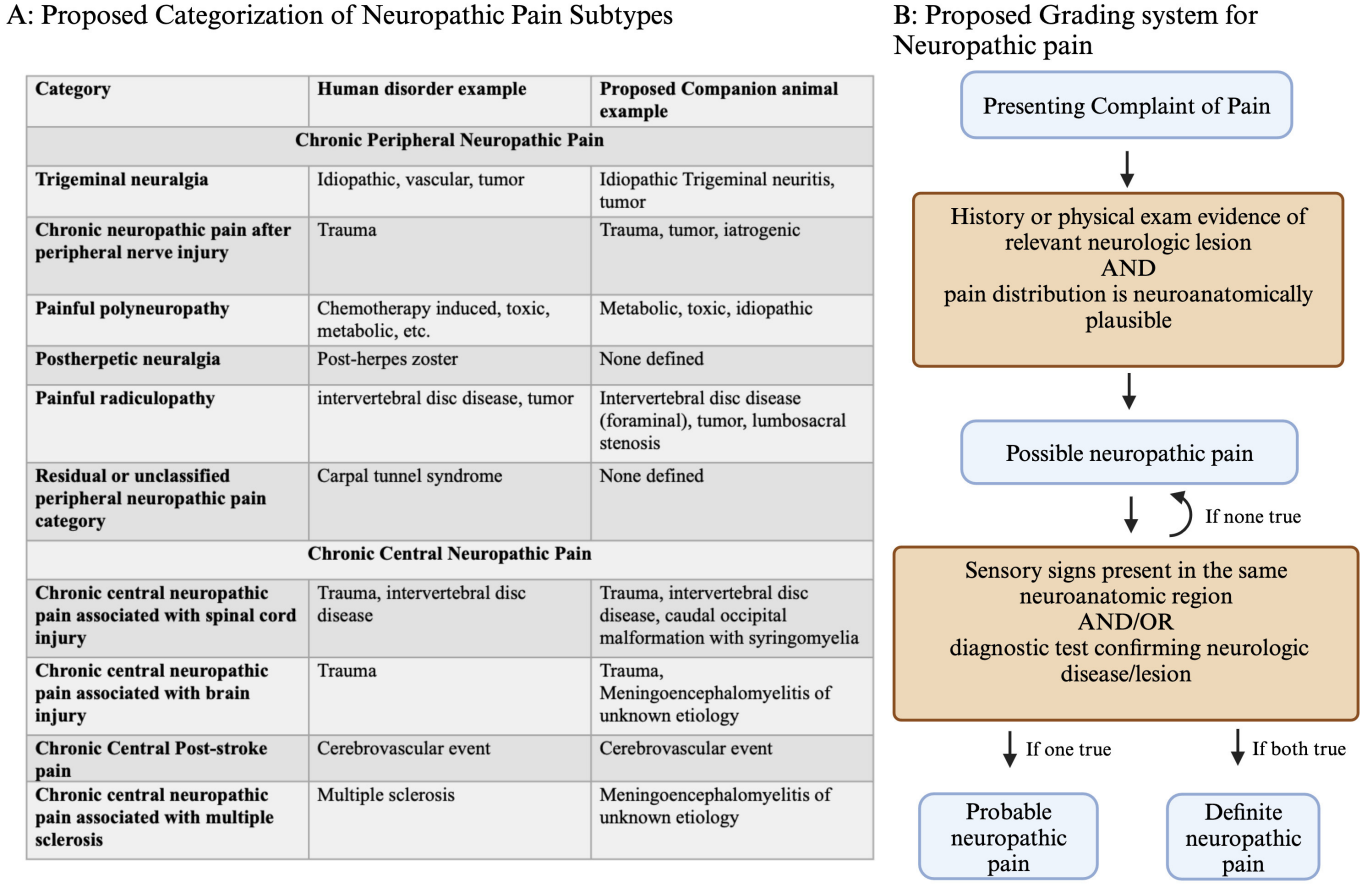
**(A)** Classification of neuropathic pain, including central and peripheral subtypes, with corresponding examples from humans and proposed corresponding neuropathic pain disorders in companion animals. **(B)** Grading system decision tree for confidence in neuropathic pain diagnosis. For patients presenting with pain, a history of relevant neurologic lesions and a matching distribution is consistent with possible neuropathic pain. The presence of either sensory signs (loss of sensation or increased/altered sensation) or lesion confirmation based on diagnostics results in the conclusion of probable or definite neuropathic pain. Created with BioRender.com.

The perception of pain, or nociception, is normally a protective mechanism. However, chronic pain can also be a maladaptive pathologic disorder (10). Chronic pain of neurologic origin is classified as either peripheral or central in humans and then is further characterized by origin and mechanism (Figure 1A) (6). For neuropathic pain, no similar classification has been established in companion animals. By utilizing this classification scheme and applying it to companion animals, we may improve our knowledge of neuropathic pain in these species.

The neuropathic pain grading system captures information about the probability that neuropathic pain is present, using possible, probable, or definite based on the clinical picture (Figure 1B) (11, 12). This type of classification scheme was originally proposed in recognition of the lack of a universal method to diagnose neuropathic pain, which is a continued problem in veterinary and human medicine (12). Applying these grading criteria to companion animals can capture a clinician's level of certainty that neuropathic pain is present, which may guide treatment. Recording neuropathic pain grade may be useful for deciding when to initiate treatment, determining treatment effectiveness, or documenting information for future retrospective studies.

For each of the major neuropathic pain categories that are described in humans, examples of similar companion animal disorders are shown (Figure 1A). One cause of neuropathic pain in companion animals is caudal occipital malformation (CM) with syringomyelia (SM), which is a chronic central neuropathic pain associated with spinal cord injury (4, 7, 8). Another example of neuropathic pain is degenerative lumbosacral stenosis, which is a painful radiculopathy (13, 14). However, some sources of neuropathic pain in companion animals are under-recognized, such as poststroke pain or painful polyneuropathies. Applying the human classification and grading of neuropathic pain to companion animals may improve the recognition, diagnosis, and treatment of these disorders.

## 3 Physiology of nociception

**Figure 2:**
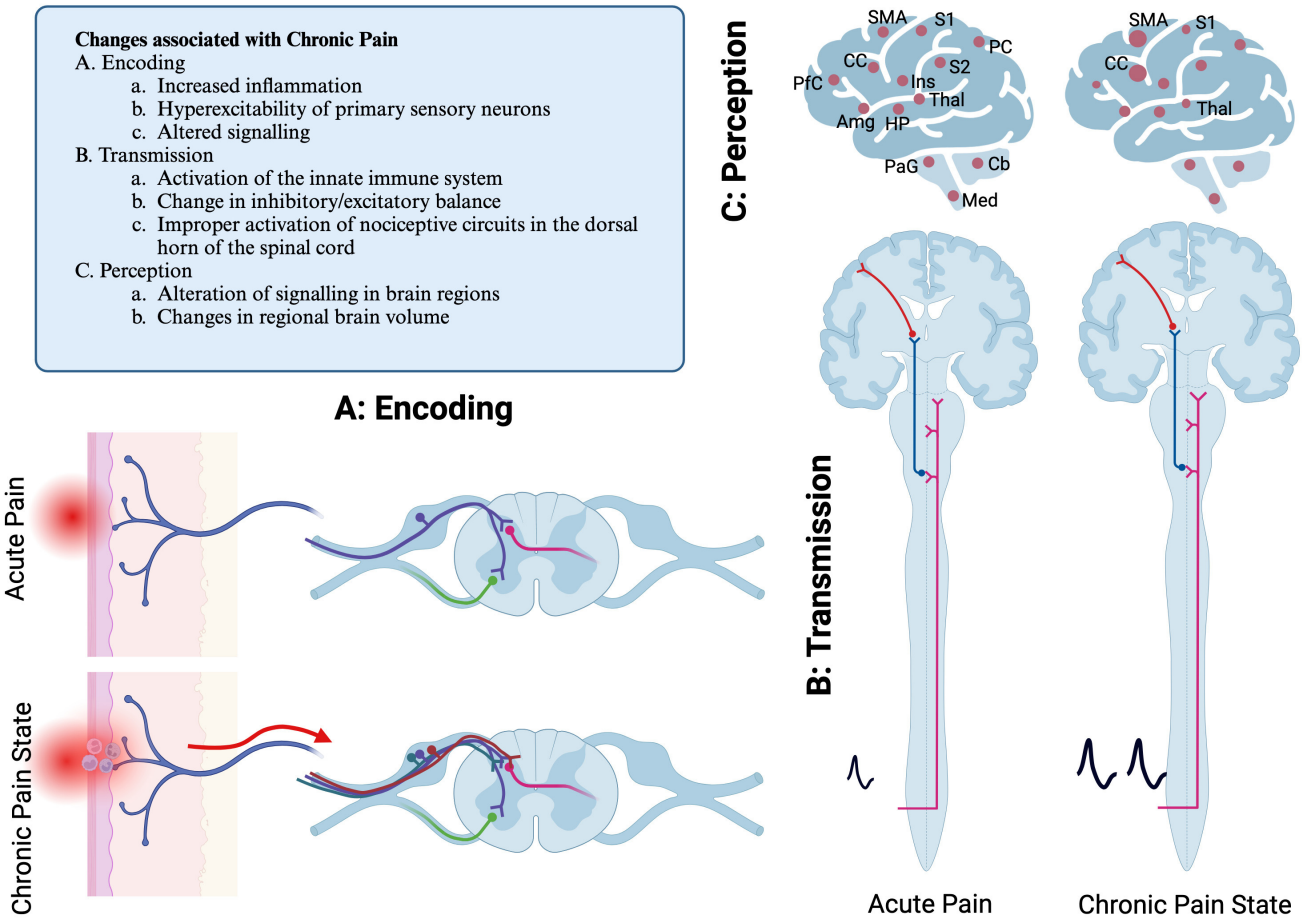
Schematic diagram of pain pathways showing primary location of reflexive and ascending pain pathways. Mechanisms of chronic pain development are illustrated as well. **(A)** Encoding step, illustrating a free nerve ending that is activated due to acutely painful stimulus. **(B)** Transmission, the spinothalamic tract is pictured. Not pictured are other sensory pathways, including dorsal column postsynaptic pathway, the spinocervicothalamic pathway, the spinomesencephalic pathway and quintothalamic pathways. **(C)** Illustration of the regions of the brain that are associated with painful stimuli. These may have sensory or affective valence. In chronic pain, regions may either be differentially active, or there can be changes in brain volume. S1, primary sensory cortex; S2, secondary sensory cortex; Thal, thalamus; Ins, insula; PC, parietal cortex; CC, cingulate cortex; PfC, Prefrontal cortex; HP, hippocampus; Amg, amygdala; PaG, periacqueductal gray; Cb, Cerebellum; Med, Medulla. Created with BioRender.com.

The pathways for sensing and processing nociceptive information are part of the “pain, touch, and temperature” system that make up the general somatic afferent systems (GSA) (15). Nociceptive stimuli, such as mechanical or thermal stimuli, are encoded by activation of nerve endings, which may be located in the skin, deep tissues, or organs (16). The most common nerve endings to detect nociceptive signals are free nerve endings, though other cells such as epithelial cells and Merkel cells may contribute to the initial encoding step (16). The cell bodies of these pseudounipolar primary sensory neurons form the dorsal root (DRG) and trigeminal ganglia (TG) (Figure 2A). Primary sensory neurons are classified by the type of information they transmit, axon size, myelination, and conduction velocity. Other classification systems have also been proposed based on gene expression patterns or electrophysiologic properties (16, 17).

The axons from the DRG enter the dorsal horn of the spinal cord via the dorsal root and synapse in the superficial layers of the spinal cord (Figure 2A) (15–17). Nociceptive information from the spinal cord is transmitted to reflexive pathways, local processing occurs, and information is transmitted to the brain. The primary nociceptive pathway in humans is referred to as the spinothalamic tract. However, in companion animals, important nociceptive pathways also include the dorsal column postsynaptic pathway, the spinocervicothalamic pathway, and the spinomesencephalic pathway (15). Nociceptive information from the head is primarily encoded by sensory neurons in the trigeminal nerve. Therefore, the majority of sensory information from the head is transmitted in the quintothalamic pathway. These pathways transmit nociceptive information to several locations in the brain. Together, the GSA system in companion animals can be called the spinothalamic system, though that terminology does not fully capture the myriad pathways of nociceptive transmission (Figure 2B) (15).

While much of the nociceptive information is transmitted via the spinothalamic tract to the primary sensory cortex via the thalamus, several other areas of the brain respond to an acute nociceptive stimulus. These include the insular cortex, cingulate cortex, prefrontal cortex, posterior parietal cortex, the secondary somatosensory cortex, the amygdala, hippocampus, and motor cortex (16, 18, 19). Other areas include the cerebellum, medulla, and periaqueductal gray region. Some of these regions, such as the somatosensory cortex, thalamus, and insular cortex, are important for the sensory aspects of pain, while the cingulate cortex, insular cortex, and prefrontal cortex process the affective aspects of pain (18). The regions of the brain that respond to painful stimuli are sometimes collectively referred to as the Pain Matrix. This concept emphasizes the complexity of processing of nociceptive information.

## 4 Pathophysiology of chronic pain

Alterations in the cellular and network processing of nociception are thought to underly the development of chronic pain. It may take days to weeks for chronic pain to develop, and these alterations in neuronal function often persist even after the originating tissue damage has resolved (11, 20). These maladaptive responses occur at several levels in the nociceptive network, including encoding, transmission, and perception (Figure 2).

In chronic pain states, inflammation or injury to the primary sensory neurons results in hyperexcitability and increased firing. Other possible causes of pain may include an imbalance between ascending and descending signaling pathways (20). Chronic pain can result in changes in gene transcription and translation in individual neurons and support/glial cells in the dorsal root ganglia and spinal cord (21). This may cause altered processing of sensory information in the spinal cord. Additionally, changes in the brain's response to painful stimuli also occur, which can be measured as difference in regional blood flow (16, 18, 19). Studies have identified changes in brain volume, including loss of volume in the primary somatosensory cortex and thalamic gray matter or increased tissue volume in the cingulate cortex and primary motor cortex (18, 20).

## 5 Diagnosis of pain in companion animal clinical practice

The diagnosis of pain for companion animals currently relies on owner reporting, physical examination findings, and direct observation of the pet. The diagnosis of pain is complicated by the inherent limitations of examining animals in the veterinary setting. The stress of a hospital visit may mask subtle aspects of pain the owner may appreciate in a home setting. The role of veterinary visits in anxiety is not well understood (22). The clinician sometimes must rely upon other factors, including patient signalment or owner reports or videos.

Observational findings may suggest the presence of pain, including changes in posture, facial expression, gait, or tone. During the physical and neurologic examinations, other indicators of pain may include heart rate, respiratory rate, or muscle atrophy. Pain or muscle fasciculations may be elicited on palpation. However, not all animals will respond to palpation, and it can be difficult to localize pain. Additionally, in referral settings, many patients have previously received pain medication, which can mask the clinical manifestations of pain.

Structured assessments, such as owner questionnaires for pain, include the Canine Brief Pain Inventory (CBPI), Neuropathic Pain scoring (NeP), and quality of life visual analog scoring (VAS) (4, 23, 24). These are short questionnaires that can be repeatedly filled out by owners over time. However, they are underutilized in practice. Additionally, these forms may rely on animals having a clinical diagnosis, as the questions can be biased toward specific diagnoses, as with the NeP questionnaire. There is little evidence that one survey is the most accurate or effective, and it can be difficult to compare responses across questionnaires.

Clinician scoring systems may be useful, particularly in the context of acute pain. The two most common are the Colorado acute pain score and the Modified Glasgow scale (25–27). Behavioral scales have been developed for cats as well (28). Facial grimace has been validated as a measurement of pain in cats (29, 30). Limitations of these assessments include their reliance on user experience, lack of consideration for patient anxiety, and lack of validation for chronic pain (27, 31).

Studies have validated quantitative measurements of pain, primarily for clinical research. Quantitative sensory testing (QST) has been utilized to test allodynia and hyperalgesia in the context of musculoskeletal and neuropathic pain (32–34). These methods include Von Frey anesthesiometry and cold latency to measure a patient's responses (35–37). These QST techniques can be challenging to administer reliably and therefore may need to be validated by each user (38). Additionally, QST outcome measures are not specific for pain type such as musculoskeletal vs. neuropathic pain and therefore cannot be used in isolation as a measure of neuropathic pain (9, 32).

Actigraphy or accelerometry collars can be used to analyze canine behavior, including to measure aspects of pain (39, 40). In some cases, a simple step count may be useful (40). However, some authors have found there is not a strong correlation between the number of steps and musculoskeletal pain. This may be due to the relationship between an owner's activity and a pet's behavior.

Imaging and electrophysiologic techniques have also been used for pain detection in dogs and cats. For example, in dogs with SM, the location, size, and distribution of the syrinx, as visualized by MRI, can predict the presence of pain (41). In degenerative lumbosacral stenosis, electrodiagnostics, such as F-waves or cord dorsum potentials, may be useful for detecting dogs with painful radiculopathies and confirming a neuropathic pain diagnosis (42).

## 6 Current research gaps and opportunities

Utilizing technology such as video cameras, high-speed internet, and collar accelerometer trackers, we may be able to incorporate information and observations from the animal's daily environment into clinical practice. This is an active area of study in musculoskeletal research, but there are limited studies in neuropathic pain thus far (40, 43, 44). Understanding similarities and differences between behaviors at home and clinical observations utilizing these technologies would be a meaningful first step toward understanding their utility for diagnosing neuropathic pain in companion animals.

The relationship between sleep and pain is of interest in humans (see below) as poor sleep and progression of chronic pain are linked (45, 46). Several recent studies have shown effective methods to measure sleep in dogs. This includes developing questionnaires for owners and simplified methods to place electrodes for polysomnography (47, 48).

Although QST is fairly well described, this group of methods currently has limitations due to the high interobserver variabliity, and the variety of techniques used to perform the QST (32, 35, 36, 38, 49). Improving methods of QST and making them more accessible would be beneficial.

Imaging techniques have already been applied to diagnose pain in dogs with SM, where specific imaging features are correlated with chronic pain (50). However, it is difficult to correlate the degree of pain and relevant imaging findings in dogs with degenerative lumbosacral stenosis or intervertebral disc disease, though these both frequently cause neuropathic pain. Perhaps imaging the brain of animals with neuropathic pain may be useful, even if the brain is not considered the primary source of the pain. In humans, chronic pain is correlated with changes in specifics areas of the pain matrix regions of the brain.

## 7 Cross-species comparisons and opportunities

Over 20% of the US human population experiences chronic pain (51). Chronic pain is defined as pain that occurs either most days or every day and lasting 3 months or longer. The diagnosis of chronic pain in human medicine also relies on a through history including duration of pain, historical injuries, or previous painful episodes. A visual analog scale or numerical score is solicited to determine the perceived severity of pain, although the perception of pain is a subjective measure that differs between individuals (52). The clinician will also question the human patient as to the characteristics of pain, such as tingling, sharp pain, numbness, or burning.

The medical community has recognized the importance of biopsychosocial factors of pain in humans (53). This indicates that the experience and impact of pain in humans is not simply related to tissue trauma. Screening for other psychosocial factors, such as coping behaviors, drug addiction, social support, sleep quality/disorders, and environment, is performed (54). The presence of these risk factors may affect the risk for developing chronic pain as well as the response to treatment. One specific example of a biopsychosocial factor from humans that could be studied in dogs is sleep. Sleep abnormalities are correlated with chronic pain, and insomnia is common (46). Interestingly, sleep impairment is also predictive of worsening chronic pain over time (45). A better understanding of the relationship between sleep and pain in animal could aid in monitoring chronic pain.

Rodents are the most commonly used model of pain for translational research. They are utilized for research into the pathophysiology and treatment of pain. The advantages of rodents are significant and include accessibility to genetic manipulation and repeatable pain models. Similar to humans, there are social and behavioral factors in rodents that affect pain behaviors, such as social housing, stress, or being housed with other animals that are in pain (55). These factors may increase (hyperalgesia) or in some cases decrease a pain response, in the case of stress-induced analgesia (55). The method of testing may also affect pain responses. Factors such as habituation time and handler experience are also important in pain testing. Testing for pain in clinical practice is subject to variables that are often outside of our control, such as transportation or being handled by strangers. However, considering these social factors in pets is likely important when trying to grade and localize neuropathic pain.

The measurement of pain in rodents is accomplished in several ways, such as Von Frey Filament testing and temperature testing. Recent methods of video analysis in rodents have altered the way that we think about pain measurement, as this has helped us expand from reflexive or evoked pain behaviors (of which QST is an example) to also studying spontaneous behaviors. By quantitatively evaluating spontaneous behaviors, we may better understand pain behaviors and response to analgesics (57, 60). Specific behavioral responses such as the trajectory of the paw during withdrawal assays in mice are strain specific, and this type of behavioral analysis may be interesting to study pain responses in different breeds of dogs (56).

## 8 Discussion

There are opportunities for improving the diagnosis and treatment of chronic neuropathic pain in dogs and cats. When we study the well-defined categories of neuropathic pain in humans, there are some categories that are easily recognized by veterinarians, such as painful radiculopathies and chronic central neuropathic pain associated with spinal cord injury. However, other categories of neuropathic pain reported in humans are not well understood in veterinary species. It is important to recognize that other categories of neuropathic pain, for example disorders that causes painful polyneuropathies, may be present in our veterinary patients. Currently, we may miss some forms of neuropathic pain. For example, if animals are experiencing paresthesias or dysesthesias that cause tingling or numbness, we as clinicians may fail to recognize those animals as experiencing neuropathic pain with any of the currently available metrics.

Priorities for improved pain measurement include improving the detection of pain in the context of anxiety, the stress of veterinary visits, other behavioral changes, or dysphoria. It is interesting to consider that anxiety or stress may be associated with chronic pain in humans while it may mask the diagnosis of chronic pain in companion animals.

We likely need to develop multiple new methods to measure chronic pain and neuropathic pain, as there is a diversity of causes and locations. Ideally, these methods will have high sensitivity, with the ability to discriminate painful and non-painful animals in a clinically useful manner, and each must be carefully validated. Some opportunities to improve chronic pain detection include owner questionnaires, behavioral analysis through video or actigraphy, advanced imaging, and electrodiagnostic techniques. Ultimately, we need methods that are easy to implement into clinical practice, so that they can be applied to our dog and cat patients and identify patients that would benefit from treatment.
